# The mycotoxin alternariol suppresses lipopolysaccharide-induced inflammation in THP-1 derived macrophages targeting the NF-κB signalling pathway

**DOI:** 10.1007/s00204-018-2299-4

**Published:** 2018-09-03

**Authors:** Jessica Kollarova, Ebru Cenk, Cornelia Schmutz, Doris Marko

**Affiliations:** Department of Food Chemistry and Toxicology, Faculty of Chemistry, University of Vienna, Waehringerstr. 38, 1090 Vienna, Austria

**Keywords:** Mycotoxin, Alternariol, miRNA, NF-κB, Immune response

## Abstract

Alternariol (AOH) is a secondary metabolite formed by black mold of the genus *Alternaria alternata*. Due to limited hazard and occurrence data, AOH is still considered as an “emerging mycotoxin” and, as such, not monitored and regulated yet. Recent studies indicate immunosuppressive effects in vitro by altering the expression of CD molecules and proinflammatory cytokines, which are indispensable in mounting an innate immune response. However, the mode of action by which AOH exerts its immunosuppressive effects has not been unraveled yet. The present study aimed to characterise the impact of AOH on the nuclear factor kappa B (NF-κB) pathway, the expression of NF-κB target cytokines and involved regulatory microRNAs (miRNAs). In THP-1 derived macrophages, AOH (1–20 µM) was found to suppress lipopolysaccharide (LPS)-induced NF-κB pathway activation, decrease secretion of the proinflammatory cytokines IL-8, IL-6, TNF-α and to induce secretion of the anti-inflammatory IL-10. Thereby, a distinct pattern of cytokine mRNA levels was monitored, varying between short- and long-term exposure. Concomitantly, AOH (2–20 µM) affected the transcription levels of miR-146a and miR-155 in LPS-stimulated THP-1 derived macrophages dose-dependently by down- and upregulation, respectively. In contrast, transcription of miR-16 and miR-125b, two other immune-related miRNAs, was not modulated. In the absence of a LPS stimulus, AOH (20 µM) did not affect basal NF-κB activity, but increased IL-10 transcription. Collectively, our results indicate, that AOH itself does not induce a proinflammatory immune response in human macrophages; however, in an inflamed environment it possesses the ability to repress inflammation by targeting the NF-κB signalling pathway and regulatory miRNAs.

## Introduction

The immune system is an integral component within an organism responsible for the defense against disease and harmful influences. It comprises a variety of immune cells and can be subdivided into innate and adaptive immune system (Medzhitov and Janeway 1997). The cells of the innate immune system are the leading guardians providing an immediate non-specific defense against pathogens which is followed by the activation of the more specific and acquired adaptive immune system. Macrophages are white blood cells originating from circulating blood monocytes, residing in loci where accumulation of foreign substances is likely (e.g., intestine, liver, kidney) (Smith et al. 2005). They belong to the innate immune system and are important initiators of the specific adaptive immune response (Medzhitov and Janeway 1997). At the onset of an immune response, immune cells employ a very sophisticated way of communication by secreting various inflammatory mediators such as cytokines, eicosanoids and vasoactive amines. Gene expression of cytokines is closely related to the activation of nuclear factor kappa B (NF-κB), a transcription factor involved in inflammatory processes (Newton and Dixit 2012). Various endo- and exogenous stimuli including reactive oxygen species (ROS), viral and bacterial antigens such as lipopolysaccharide (LPS) induce a downstream signalling cascade through stimulation of various receptors [e.g., toll-like receptors (TLRs)] leading to the activation of NF-κB (Liu et al. 2017; Morgan and Liu 2011). Once activated, NF-κB is translocated into the nucleus, where it binds to the promotor region of its target genes and finally induces transcription of several inflammatory mediators (Liu et al. 2017). Cytokines, such as IL-8, IL-6 and TNF-α are expressed in a NF-κB-dependent manner and function primarily as proinflammatory signals (Tak and Firestein 2001). Inflammatory processes, however, require being controlled and regulated, thus anti-inflammatory cytokines such as IL-10 are also expressed upon NF-κB activation resulting in a complex interplay of these cytokines (Saraiva et al. 2005; Saraiva and O’Garra 2010).

Besides glucocorticoids (e.g., dexamethasone) that are known to inhibit inflammatory processes by targeting NF-κB, in recent years a vast number of naturally occurring compounds (e.g., polyphenols, mycotoxins, isothiocyanates) was also found to possess potential immunosuppressive effects (Kundu et al. 2006; Moon et al. 2009; Smoak and Cidlowski 2004; Solhaug et al. 2016). Mycotoxins are naturally occurring secondary metabolites produced by a variety of fungal species that contaminate food and feed commodities and thus, might pose a risk to human and animal health (EFSA 2011). The fungal toxin alternariol (AOH), formed by *Alternaria alternata*, has been reported to possess genotoxic, cytotoxic and estrogenic potential as well as to induce oxidative stress, autophagy and senescence in vitro (Fehr et al. 2009; Lehmann et al. 2006; Solhaug et al. 2014; Tiessen et al. 2013). Recently, dietary exposure data of AOH revealed, that the chronic dietary exposure of 1.0–15.2 ng/kg b.w. per day for adults by far exceeds the threshold of toxicological concern (TTC) value for AOH (2.5 ng/kg b.w. per day) (EFSA 2011, 2016). Consequently, EFSA emphasized the necessity of additional data to characterise a potential hazard arising from AOH exposure (EFSA 2016). Moreover, recent studies reported AOH to exhibit immunosuppressive properties in vitro by modifying the expression of CD receptors and proinflammatory cytokines (Grover and Lawrence 2017; Solhaug et al. 2016). More specifically, AOH impaired the differentiation from monocytes to macrophages by reducing the expression of CD14 and CD11b. These cell surface molecules are co-receptors of TLR4, which are important in engaging an appropriate response to bacterial endotoxins (e.g., LPS) (Zhou et al. 2005). Therefore, a reduced macrophage differentiation during inflammation might generate serious losses in the hosts’ immune system, depleting the implementation of an effective immune response. Additionally, Solhaug et al. found a reduced response of THP-1 derived macrophages to LPS after exposure to AOH, characterised by reduced secretion and transcription of the proinflammatory cytokine TNF-α (Solhaug et al. 2016). AOH has been reported to reduce gene expression of proinflammatory cytokines (IL-8, IL-6) also in other cell models (RAW264.7 macrophages and BEAS-2B human bronchial lung epithelial cells); however, the underlying mechanisms responsible for the immunosuppressive effects induced by AOH have not been identified so far (Grover and Lawrence 2017). Thus, in the present work we aimed to elucidate the mode of action of AOH by identifying potential targets responsible for its immunoregulatory effects.

In recent years, microRNAs (miRNAs) emerged as important regulators of various biological processes including cell differentiation, proliferation, apoptosis and immune response. miRNAs are small, non-coding sequences that exert their main function as post-transcriptional modificators regulating the expression of several proteins (Lee et al. 2014). Recent studies suggest a link between AOH-induced increase of the tumor suppressor protein p53 and the involvement of miR-29a and the miR-34 family (Solhaug et al. 2012; Vejdovszky et al. 2017b). miRNAs have also been reported to modulate immune responses by regulating the expression of receptors and transcription factors involved in TLR/NF-κB signalling leading to modified gene expression of target genes (e.g., cytokines) (O’Neill et al. 2011). Until now, however, the impact of AOH on non-coding RNAs that are involved in regulating the immune response is largely unexplored.

In the present study, we addressed the question whether AOH affects the NF-κB signalling pathway resulting in an altered gene expression profile of the proinflammatory cytokines IL-8, IL-6, TNF-α and the anti-inflammatory IL-10. In an exploratory approach, the role of regulatory miRNAs, that are potentially involved in AOH-induced immunoregulation, was investigated. The experiments were carried out in differentiated LPS stimulated and non-stimulated macrophages derived from the human monocytic cell line THP-1, a well-characterised cell model to study immunomodulatory effects.

## Materials and methods

### Materials

Alternariol (AOH) (high purity grade, approximately 96%) was purchased from Sigma-Aldrich Chemie GmbH (Steinheim, DE) and dimethylsulfoxid (DMSO) ≥ 99.5% from Carl Roth GmbH&Co. (Karlsruhe, DE). Phorbol 12-myristate 13-acetate (PMA) ≥ 99%, powder, lipopolysaccharide (LPS) (from *E.coli*) and dexamethasone, powder ≥ 97% were purchased from Sigma-Aldrich Chemie GmbH (Steinheim, DE). Cell culture media (RPMI-1640 with l-glutamine, RPMI-1640 with 2 mM l-glutamine, 25 mM HEPES) and supplements (penicillin–streptomycin (P/S) 100 U/ml, fetal bovine serum (FBS)) were purchased from Invitrogen™ Life Technologies (Karlsruhe, DE) and Thermo Fisher Scientific (Darmstadt, DE). Normocin and zeocin were purchased from Invivogen (CA, US). AlamarBlue™ Cell Viability Reagent was purchased from Thermo Fisher Scientific (Darmstadt, DE) and Quanti-Luc™ from Invivogen (CA, US). All primers (GAPDH, ACTB, CXCL8, TNF-α, IL-10, IL-6, SNORD68, RNU6, miR-146a, miR-155, miR-125b, miR-16), QIAzol Lysis Reagent and all kits (QuantiTect SYBR Green PCR Kit, RNeasy Mini Kit, miRNeasy Mini Kit, QuantiTect Reverse Transcription Kit, miScript II RT Kit, RNase free DNase Set) used for qRT-PCR were purchased from Qiagen (Hilden, DE). ProcartaPlex Mix&Match Human 4-plex was purchased from Invitrogen™ Life Technologies (Karlsruhe, DE).

### Cell culture and treatments

The human monocytic cell line THP-1 (ATCC, US) and THP1-Lucia™ NF-κB monocytes (Invivogen, US) (used for monitoring the NF-κB activity) were maintained in RPMI-1640 medium with either l-glutamine (THP-1 monocytes) or with 2 mM l-glutamine, 25 mM HEPES (THP1-Lucia™ NF-κB monocytes). Both media were furthermore supplemented with heat-inactivated 10% FBS and 1% P/S (100 U/ml). Monocytes were subcultured every third to fourth day to maintain cell concentration below 1 × 10^6^ cells/ml and they were kept in humidified incubators at 37 °C and 5% CO_2_. Antibiotic formulations zeocin and normocin (100 µg/ml) were furthermore alternately added to THP1-Lucia™ NF-κB monocytes every second time of subculturing. AOH and dexamethasone were dissolved in DMSO, whereas LPS in cell culture media. Appropriate amounts of solvent were included into all controls, differentiated monocytes were therefore exposed to a final concentration of 0.1% DMSO.

For all assays, THP-1 monocytes were seeded (4.2 × 10^5^ cells/well) in 6-well plates and differentiated into adherent macrophages with 10 ng/ml PMA for 72 h. Cells were kept in the incubator for another 24 h in PMA-free media until incubation. Dexamethasone (1 µM) and LPS (10 ng/ml) served as positive controls.

### Alamar blue assay

After seeding (4.2 × 10^5^ cells/well) and differentiating THP-1 monocytes in 6-well plates, macrophages were preincubated with AOH (0.02–20 µM), dexamethasone (1 µM) and solvent control (0.1%) for 2 h followed by LPS (10 ng/ml) treatment for further 3 or 18 h. After incubation, macrophages were washed with PBS (37 °C) and reconstituted with fresh P/S and FBS free media (1 ml/well). Metabolic activity was measured with Alamar Blue Cell Viability Reagent according to manufacturer’s protocol (Thermo Fisher Scientific). The resulting fluorescence intensity from metabolically active cells was measured using a microplate photometer at 530 nm excitation and 560 nm emission.

### NF-κB reporter gene assay

THP1-Lucia™ NF-κB monocytes were seeded (4.2 × 10^5^ cells/well) and differentiated in 6-well plates for 72 h. After another 24 h in PMA-free media differentiated monocytes were preincubated with AOH (0.02–20 µM), dexamethasone (1 µM) and solvent control (0.1%) for 2 h followed by LPS (10 ng/ml) treatment for further 18 h. Following treatment, cell supernatant was collected and the reporter gene assay was performed according to the manufacturer’s protocol using a coelenterazine-based luminescence assay reagent, Quanti-Luc™ (Invivogen). NF-κB activation was determined by measuring luciferase activity in a microplate reader.

### qRT-PCR

THP-1 monocytes were seeded (4.2 × 10^5^ cells/well) in 6-well plates and differentiated into adherent macrophages prior to incubation. Macrophages were preincubated for 2 h with AOH (0.02–20 µM), dexamethasone (1 µM) or solvent control (0.1%) followed by LPS (10 ng/ml) treatment for further 3 or 18 h. qPCR was performed to measure gene transcription levels of IL-8, IL-6, TNF-α, IL-10 and miR-16, miR-125b, miR-146a and miR-155. Total RNA was isolated by following manufacturer’s instructions either using RNeasy Mini Kit (for cytokine gene transcription) or miRNeasy Mini Kit (for miRNA gene transcription) (Qiagen). RNA purity and concentration were determined using NanoDrop 2000C. Thereafter, isolated RNA was reverse transcribed to complementary DNA (cDNA) according to the manufacturer’s protocol (QuantiTect Reverse Transcription Kit and miScript II RT Kit, Qiagen). Subsequently, qPCR was performed using QuantiTect SYBR Green PCR Kit (Qiagen) and specific primers for IL-8, IL-6, TNF-α, IL-10 and miR-16, miR-125b, miR-146a, miR-155 (QuantiTect Primer Assay, Qiagen). All reactions were carried out at 20 µl volume and for gene transcription normalisation of cytokines housekeeping genes ß-actin and GAPDH were used, for miRNA transcript analysis SNORD68 and RNU6 were utilized.

All qPCR reactions were carried out using StepOnePlus™ real time PCR system following manufacturer’s protocols, either QuantiTect^®^ SYBR^®^ Green RT-PCR Handbook (for cytokine gene transcription) or miScript PCR System Handbook (for miRNA gene transcription). Obtained qPCR data (C_*T*_ values) were analysed by the comparative ${2^{ - \Delta \Delta {{\text{C}}_T}}}$ method for relative quantification (Schmittgen and Livak 2008).

### Cytokine measurement, Procartaplex™ Multiplex Immunoassay

THP-1 monocytes were differentiated and treated as described previously in 2.4. Afterwards, the cell supernatant containing secreted proteins was collected and centrifuged (10.000×*g*, 4 °C, 15 min) to remove cell debris. Protein levels of IL-8, IL-6, TNF-α and IL-10 were determined and analysed by a bead-based immunoassay according to manufacturer’s protocol (ProcartaPlex™ Multiplex Immunoassay, Invitrogen™ Life Technologies).

### Statistical analysis

Statistical significances were calculated in Origin Pro 9.1 using one-way ANOVA with post hoc Holm–Bonferroni test and a two-sample *t* test. If not normally distributed, a non-parametric Kruskal–Wallis ANOVA with Mann–Whitney *U* test was used. All data are shown as means ± SD and values of *p* ≤ 0.05 were considered as significant.

## Results

### Cytotoxicity

**Fig. 1:**
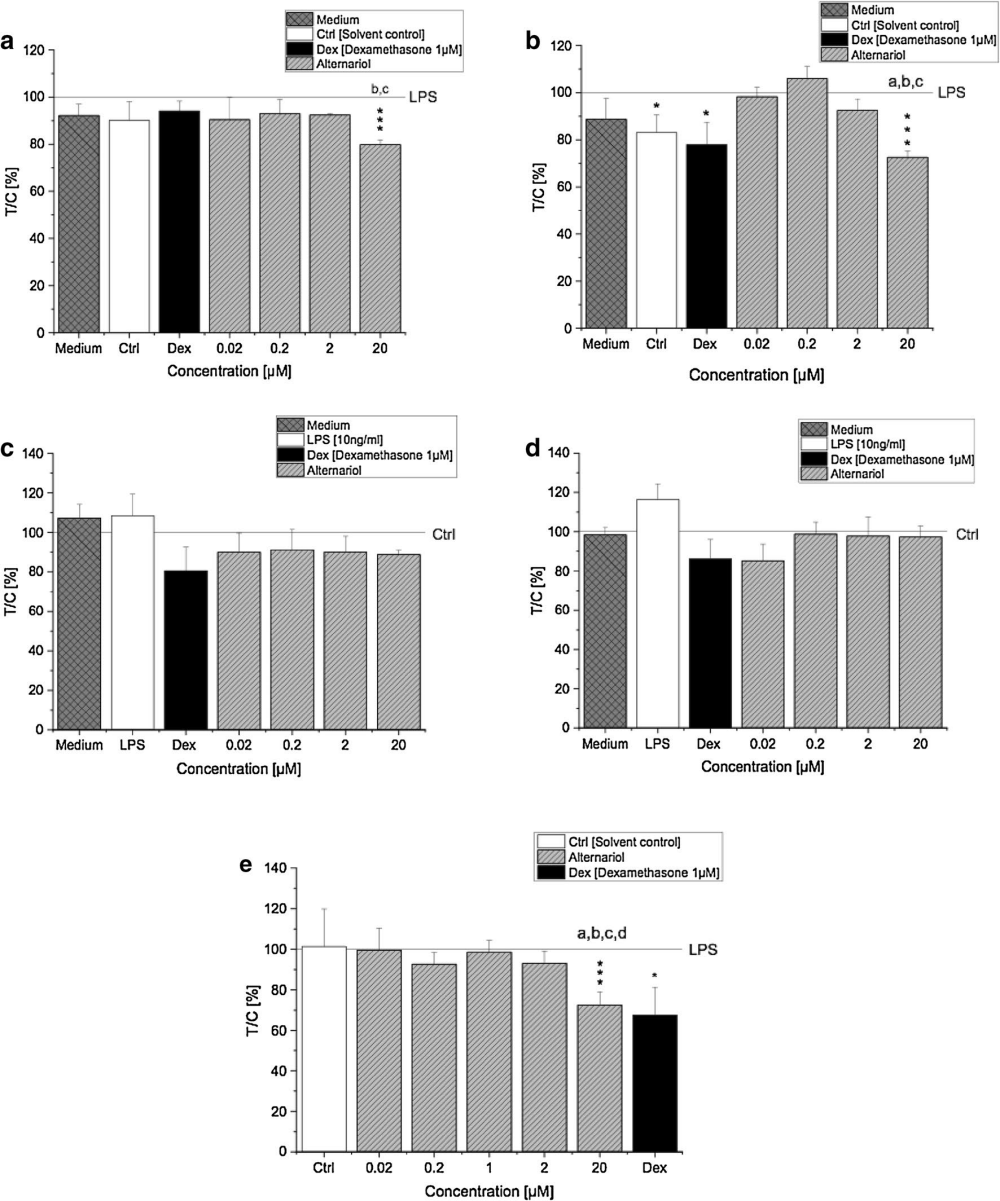
Cytotoxic effects of AOH in LPS and non-stimulated differentiated THP-1 derived macrophages measured with the Alamar Blue^®^ assay. Differentiated LPS-stimulated THP-1 cells were preincubated with AOH for 2 h and afterwards stimulated with LPS (10 ng/ml) for 3 h (**a**) and 18 h (**b**). Differentiated non-stimulated THP-1 cells were exposed to AOH for 5 h (**c**) and 20 h (**d**). THP1-Lucia™ NF-κB cells were preincubated with AOH for 2 h and stimulated with LPS for further 18 h (**e**). Fluorescence intensity was calculated as the percent of treated cells over control cells [treated with LPS or the solvent control) × 100 (*T*/*C*, %). Results are normalized to LPS or the solvent control (0.1% DMSO], respectively, expressed as mean ± SD of *T*/*C* (%). Statistical significances between varying concentrations of AOH were evaluated by one-way ANOVA and Holm–Bonferroni test (**a**–**d** *p* < 0.05), significances compared to LPS/solvent control were calculated with a two-sample *t* test (*p; **p; ***p < 0.05, 0.01, 0.001); *n* = 3–7 independent experiments

The magnitude of cytotoxicity induced by AOH after 5 and 20 h exposure was determined with the Alamar Blue assay. THP-1 cells (LPS-stimulated and non-stimulated THP-1 derived macrophages) were exposed to AOH (0.02–20 µM) and cell viability was determined by measuring the metabolic activity. Only the highest concentration of AOH (20 µM) induced significant (*p* < 0.001) cytotoxic effects in LPS-stimulated THP-1 derived macrophages at both time points (Fig. 1a, b). Furthermore, LPS-stimulated cells exposed to 20 µM AOH for 20 h showed a more pronounced decrease in viability (72.6 ± 2.6%) than cells exposed for 5 h (79.8 ± 2.0%), indicating that cytotoxicity of AOH increases over time. A comparable impact on cell viability was observed in THP1-Lucia™ NF-κB cells, which appeared most sensitive towards 20 µM AOH, showing significant (*p* < 0.001) cytotoxic effects compared to LPS (Fig. 1e). The observed reduction of viability by 20 µM AOH was however only limited. Of note, AOH did not induce cytotoxic effects in non-stimulated macrophages, since cell viability remained at a level ≥ 80% for all of the applied concentrations (Fig. 1c, d). No major changes between the respective medium controls and the solvent control (0.1% DMSO) were observed with respect to mitochondrial activity, indicating that 0.1% DMSO did not induce cytotoxicity. Therefore, experimental artifacts associated with cytotoxicity can be excluded.

### AOH suppresses LPS-induced NF-κB activation

**Fig. 2:**
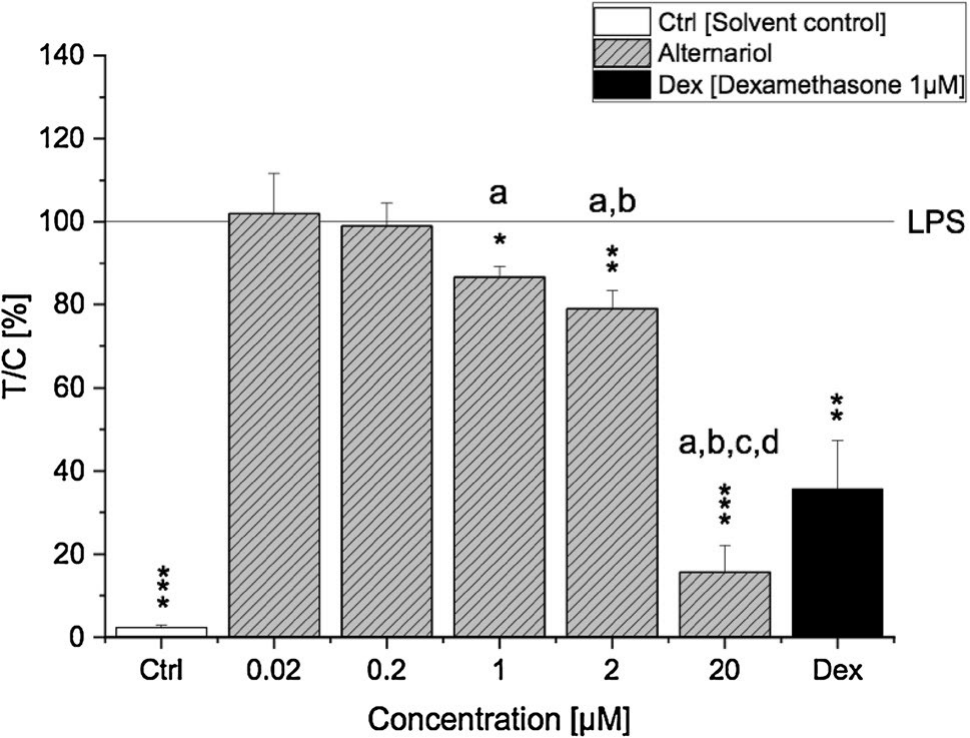
Activity of NF-κB in LPS-stimulated THP1-Lucia NF-κB cells. THP1-Lucia NF-κB cells were preincubated with AOH for 2 h followed by an 18 h LPS challenge (10 ng/ml). Luminescence intensity of the expressed luciferase protein was measured by the NF-κB reporter gene assay and calculated as the percent of treated cells over control cells (treated with LPS) × 100 (*T*/*C*, %). Results are normalized to LPS and are expressed as mean ± SD of *T*/*C* (%). Statistical significances between varying concentrations of AOH were evaluated by one-way ANOVA and Holm–Bonferroni test (**a**–**d** *p* < 0.001) and significances compared to LPS were calculated with a two-sample *t* test (*p; **p; ***p < 0.05, 0.01, 0.001); *n* = 3–6 independent experiments

Lipopolysaccharide is a ligand of TLR4 known to potently induce a proinflammatory immune response via activation of the NF-κB signalling pathway. To characterise whether AOH modulates the LPS-induced activation of the proinflammatory NF-κB signalling pathway, a NF-κB reporter gene assay was used. After exposing THP1-Lucia™ NF-κB cells to AOH (0.02–20 µM) for 2 h and stimulating them with LPS (10 ng/ml) for further 18 h, the luminescent signal intensity resulting from NF-κB activation was measured. Interestingly, preincubation with AOH suppressed an LPS-induced NF-κB activation in THP1-Lucia™-derived macrophages in a concentration-dependent manner (Fig. 2**)**. A significant (*p* < 0.05) decrease of the luminescent signal was observed already with 1 µM AOH and further enhanced by 2 µM (*p* < 0.01) and 20 µM (*p* < 0.001). Moreover, the observed suppressive effect of the highest AOH concentration (20 µM) was even more potent than the one of the positive control dexamethasone (*p* < 0.01).

### Modulation of cytokine gene transcription by AOH

**Fig. 3:**
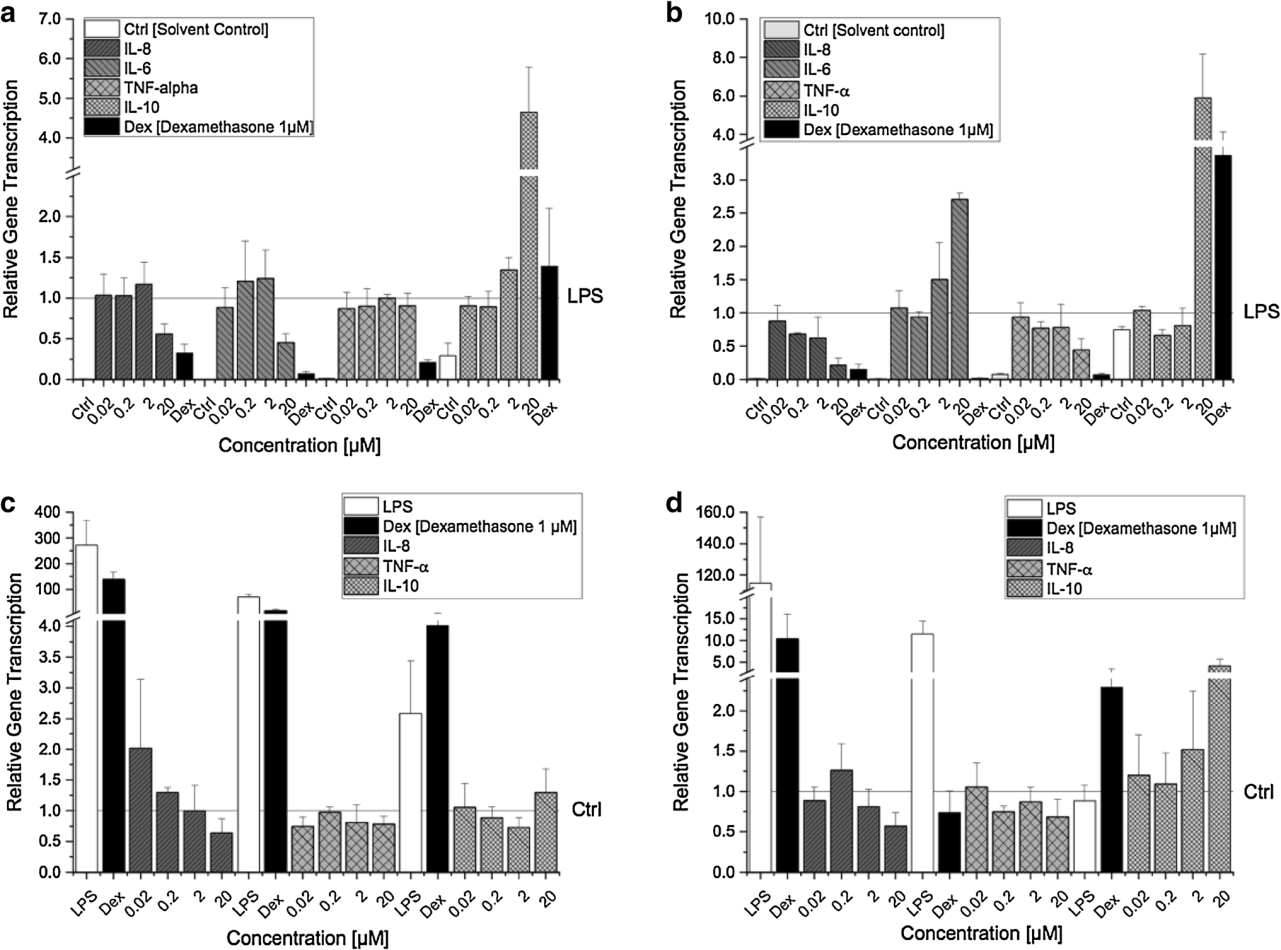
Relative gene transcription levels of IL-8, IL-6, TNF-α and IL-10 in LPS- and non-stimulated THP-1 derived macrophages after AOH exposure. Differentiated LPS-stimulated THP-1 cells were preincubated with AOH for 2 h and afterwards stimulated with LPS (10 ng/ml) for 3 h (**a**) and 18 h (**b**). Differentiated non-stimulated THP-1 cells were exposed to AOH for 5 h (**c**) and 20 h (**d**). Relative transcript levels were measured with qRT-PCR. Results are expressed as mean ± SD of the relative gene transcription (${2^{ - \Delta \Delta {{\text{C}}_T}}}$) and normalized either to LPS or the solvent control (0.1% DMSO). Statistical significances between varying concentrations of AOH were evaluated by Kruskal–Wallis ANOVA and significances compared to LPS/solvent control were calculated by Mann–Whitney *U* test; *n* = 3–7 independent experiments

Alterations of signalling cascades, including modifications of transcription factors often result in altered target gene transcription. Considering the observed suppressive effect of AOH on the proinflammatory NF-κB pathway (Fig. 2), we analysed whether this effect is also reflected on the transcriptional level. Four cytokines (IL-8, IL-6, TNF-α, IL-10), known to be affected in their expression by the NF-κB pathway were selected and the impact of AOH on the respective mRNA levels was investigated in LPS- and non-stimulated THP-1 derived macrophages. To compare the impact of short- and long-term exposure, the cells were exposed to AOH for either 5 h or 20 h (with or without LPS). After 5 h incubation, AOH significantly downregulated the LPS-induced transcription of proinflammatory cytokines IL-8 (*p* < 0.01) and IL-6 (*p* < 0.05) in a concentration-dependent manner (Fig. 3a), which is in line with the suppressive effect of AOH on LPS-induced NF-κB activation (Fig. 2). No impact on TNF-α gene transcription was observed in LPS-stimulated macrophages after 5 h incubation. However, after a prolonged exposure time of 20 h, AOH significantly (*p* < 0.05) downregulated LPS-induced TNF-α transcription (Fig. 3b**)**. Contrary to the transcription profile of IL-6 after 5 h, the impact on IL-6 mRNA transcription was inversed after 20 h, showing a significant concentration-dependent increase of mRNA levels induced by AOH (almost threefold compared to LPS) (Fig. 3b**)**.

In contrast, in non-stimulated THP-1 derived macrophages (no LPS stimulus) IL-6 mRNA was not detectable by qPCR at both time points (5 and 20 h of incubation), indicating no impact of AOH on IL-6 transcription in the absence of an inflammatory stimulus (data not shown). Under these experimental conditions, the proinflammatory cytokine TNF-α was weakly, but significantly (*p* < 0.05) decreased by 20 µM AOH after 5 h of incubation (Fig. 3c, d). However, with increasing exposure time (20 h), the decrease was not significant anymore. Additionally, AOH concentrations below 20 µM likewise did not significantly affect TNF-α transcription, showing similar relative gene transcript levels as the solvent control at both time points.

Although IL-8 mRNA levels were increased by 0.02 µM AOH after an exposure time of 5 h and were gradually decreasing in a concentration-dependent manner, no significant difference was observed between AOH and the solvent control. After 20 h incubation, IL-8 mRNA levels were still decreased by 20 µM AOH (0.56 ± 0.22); however, the decreasing effect was again not found to be significant. In non-stimulated THP-1 derived macrophages, low concentrations of AOH (0.02–2 µM) did not affect IL-8 transcription with mRNA levels in the range of the solvent control. Contrary to the proinflammatory cytokines, AOH increased mRNA levels of the anti-inflammatory IL-10 regardless of LPS stimulation. The increasing effect was time- and concentration-dependent. In LPS-stimulated THP-1 derived macrophages only 20 µM AOH significantly (*p* < 0.01) induced IL-10 transcription, up to almost fiveand sixfold after 5 and 20 h, respectively (Fig. 3a, b). No such increasing effect of IL-10 was yet observed after 5 h in unstimulated macrophages, however, after a prolonged exposure period, AOH (20 µM) significantly increased IL-10 transcription (almost fivefold compared to solvent control) (Fig. 3c, d). Concentrations below 20 µM AOH did not affect IL-10 transcription neither in LPS nor in unstimulated macrophages.

### AOH reduces proinflammatory cytokine secretion

**Fig. 4:**
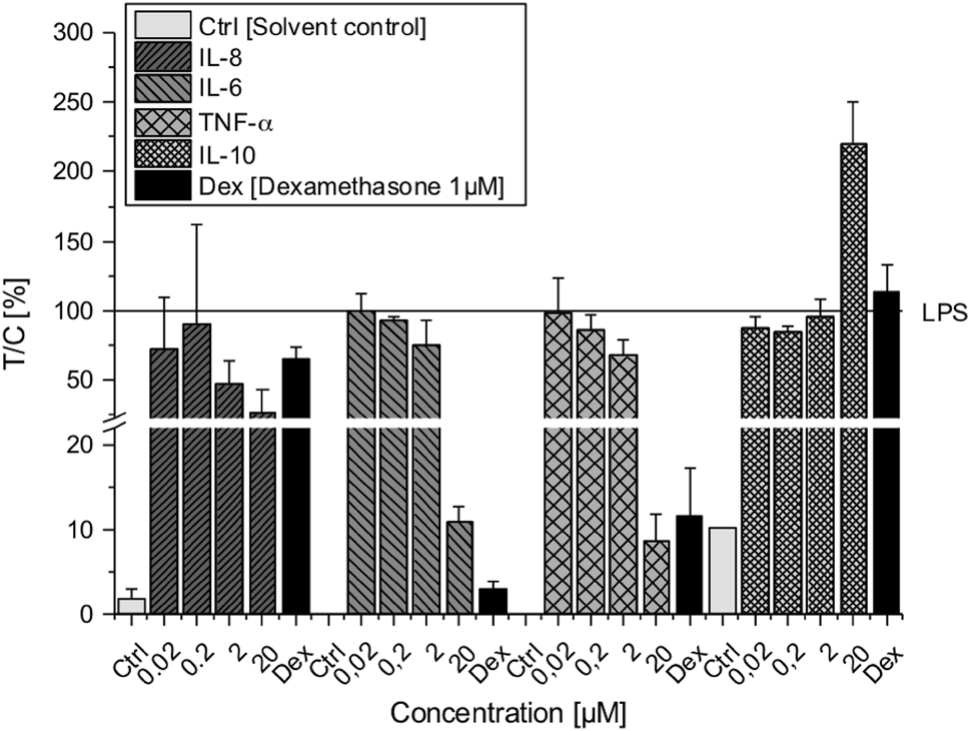
Cytokine secretion levels of IL-8, IL-6, TNF-α and IL-10 in LPS-stimulated THP-1 derived macrophages after AOH exposure measured with ProcartaPlex™ Multiplex Immunoassay. THP-1 macrophages were preincubated with AOH for 2 h followed by an 18 h LPS challenge (10 ng/ml). Cytokine protein levels were calculated as percent of treated cells over control cells (treated with LPS) × 100 (*T*/*C*, %) and are expressed as mean ± SD of *T*/*C* (%) normalized to LPS. Statistical significances between varying concentrations of AOH were evaluated by Kruskal–Wallis ANOVA and significances compared LPS were calculated by Mann–Whitney *U* test; *n* = 3 independent experiments

To evaluate to which degree previosuly measured cytokine transcript levels are reflected at protein level, we further determined cytokine secretion levels. Since no major impact of AOH was observed on cytokine transcription in non-stimulated macrophages (Fig. 3c, d), cytokine protein levels were only measured in LPS-stimulated THP-1 derived macrophages after 20 h exposure to AOH (0.02–20 µM) using a multiplex immunoassay. Protein levels of all three proinflammatory cytokines (IL-8, IL-6 and TNF-α) were found to be decreased in a concentration-dependent manner by AOH (Fig. 4). The reduction was most distinct at 20 µM AOH for all three cytokines, although reduced levels were already observed at 2 µM AOH. AOH decreased LPS-induced IL-8 (not significant) and IL-6 (*p* < 0.05) levels, which correspond to an initial suppression of the NF-κB pathway (Fig. 2) followed by reduced transcription of IL-8 and IL-6 after 5 h (Fig. 3a). AOH exposure (20 µM) furthermore significantly (*p* < 0.05) diminished LPS-induced TNF-α levels by more than 90%, indicating a potential link to the NF-κB suppression induced by AOH (Fig. 2). On the other hand, the anti-inflammatory IL-10 was significantly (*p* < 0.05) upregulated by 20 µM AOH (almost twofold compared to LPS), which is in accordance with transcript levels measured after 5 h of exposure (Fig. 3a).

### AOH modulates miRNA gene transcription

**Fig. 5:**
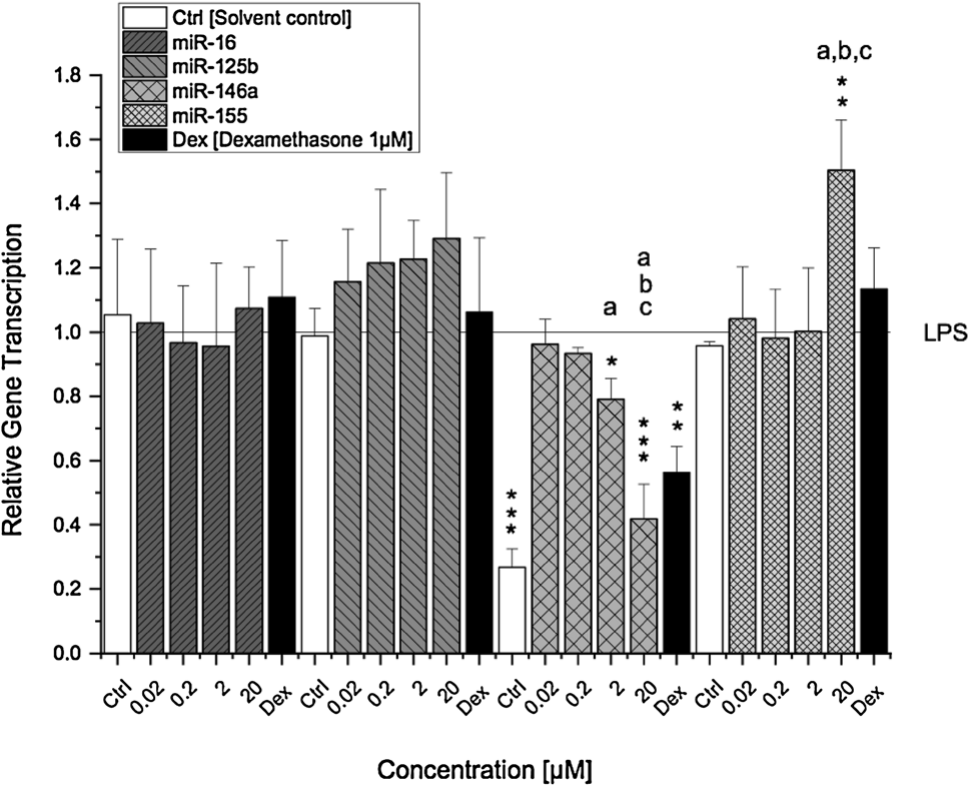
Transcription levels of miR-16, -125b, -146a and -155 in LPS-stimulated THP-1 derived macrophages after AOH exposure. THP-1 macrophages were preincubated with AOH for 2 h followed by an 18 h LPS challenge (10 ng/ml). Relative transcript levels were measured with qPCR. Results are expressed as mean ± SD of the relative gene transcription (${2^{ - \Delta \Delta {{\text{C}}_T}}}$) normalized to LPS-stimulated macrophages. Statistical significances between varying concentrations of AOH were evaluated by one-way ANOVA and Holm–Bonferroni test (**a**–**c** *p* < 0.001) and significances compared to LPS were calculated with a two-sample *t* test (*p; **p; ***p < 0.05, 0.01, 0.001); *n* = 3–5 independent experiments

The impact of AOH on miRNA transcription is illustrated in Fig. 5. Differentiated THP-1 derived macrophages were preincubated for 2 h with AOH (0.02–20 µM) and challenged with LPS for further 18 h. The relative transcript levels of miR-16, -125b, -146a and -155 were determined by a qRT-PCR and referred to LPS. Transcript levels of miR-16 and miR-125b were not significantly affected by AOH. A slight trend towards upregulation though can be observed for miR-125b in a dose-dependent manner after AOH exposure (not significant) (Fig. 5). Moreover, no differences in transcript levels of miR-16 and miR-125b were found between solvent control and LPS-stimulated macrophages. In contrast, AOH significantly reduced LPS-induced miR-146a transcript levels at a concentration of 2 µM (0.79 ± 0.06) with a further pronounced effect at 20 µM (0.41 ± 0.10). The presence of LPS did not induce miR-155 transcription; however, miR-155 was found to be significantly upregulated by 20 µM AOH (1.5-fold compared to LPS) but not by lower concentrations.

## Discussion

The present study aimed to characterise the immunosuppressive potential of AOH and its underlying mechanisms in differentiated THP-1 derived macrophages. Cytotoxicity of AOH was only marginal in LPS-stimulated THP-1 derived macrophages, which is in line with previous findings (Fig. 1a–d) (Pahlke et al. 2016; Solhaug et al. 2015, 2016; Tiessen et al. 2013). In recent times, more and more light was shed on the mycotoxin AOH and its ability to manipulate the rich network of multiple biological processes needed to strategically mount a defense response. For the first time, we report the fungal secondary metabolite AOH to exert its immunosuppressive effects via targeting the NF-κB pathway in human macrophages. The differentiation process of monocytes into active macrophages is a fundamental step towards strengthening and preparing the cells for an imminent immune response involving various phenotypic changes (e.g., expression of cell surface markers) (Yang et al. 2014). PMA activates NF-κB through inducing protein kinase C resulting in increased monocyte differentiation and expression of CD14 and CD11b surface molecules (Park et al. 2007). Previously, AOH was reported to affect PMA-induced differentiation of THP-1 monocytes by reducing the expression of CD14 and CD11b, indicating a potentially reduced immune response. Indeed, in the same study, AOH (15 µM) diminished the immune response to LPS by alleviating secretion of the proinflammatory cytokine TNF-α (Solhaug et al. 2016). Interestingly, in the present study we observed a substantial suppression of the LPS-induced NF-κB pathway activation after AOH (1–20 µM) exposure in differentiated THP-1 derived macrophages, which presumably may explain the previously observed immunosuppressive effects of AOH (Fig. 2**)**.

In several cell lines, AOH increased the levels of reactive oxygen species (ROS), which are well-known inducers of the NF-κB signalling pathway (Pahlke et al. 2016; Solhaug et al. 2012; Tiessen et al. 2013). Hence, one might suspect that cells exposed to AOH would promote ROS-induced NF-κB activation. Yet, the opposite is the case: while AOH did not affect basal NF-κB activity in THP-1 derived macrophages (experiments without LPS stimulus; data not shown), in LPS-stimulated cells AOH dose-dependently suppressed the activation of NF-κB more potently than the anti-inflammatory drug dexamethasone measured as diminished luciferase activity (Fig. 2**)**. Similar effects have been reported, e.g., for the *Fusarium* mycotoxin zearalenone (ZEN), which likewise exerts immunosuppressive effects by suppressing NF-κB activity and expression of TNF-α, yet also increasing ROS levels in vitro (Ferrer et al. 2009; Pistol et al. 2015). Thus, no direct link might exist between AOH-induced ROS disbalance and NF-κB activity.

On the other hand, the ability of AOH to act as a partial estrogen receptor (ER) agonist may, at least to some extent, be involved in its immunosuppressive action (Lehmann et al. 2006; Vejdovszky et al. 2017a). Estrogens, in particular 17ß-estradiol, have been reported to decrease LPS-induced gene expression of proinflammatory IL-6 and TNF-α in macrophages via targeting inhibitors of NF-κB signalling by miR-125b and let-7a (Deshpande et al. 1997; Murphy et al. 2010). In this respect, 17ß-estradiol was found to inhibit phosphorylation of IκBα, thus preventing nuclear translocation of NF-κB subunits. Furthermore, the expression of let-7a and miR-125b was down and upregulated, respectively, by 17ß-estradiol, thereby increasing the stability of κB-Ras2, a negative regulator of NF-κB. In comparison, Fig. 4 demonstrates that LPS-stimulated THP-1 derived macrophages exposed to AOH for 20 h also dose-dependently reduced the secretion of proinflammatory cytokines (IL-6, TNF-α and IL-8). However, in contrast to previous studies, we did not observe a significant difference in miR-125b expression between the solvent control and LPS (Fig. 5) (Tili et al. 2007). Furthermore, despite a slight increasing trend, AOH did not significantly increase miR-125b expression in THP-1 derived macrophages, indicating that AOH-induced NF-κB suppression followed by reduced secretion of IL-6, TNF-α and IL-8 is most likely mediated via a different mechanism. This disparity may be attributed to cell specificity and/or application of varying LPS concentrations (100 vs. 10 ng/ml) (Murphy et al. 2010; Tili et al. 2007).

**Fig. 6:**
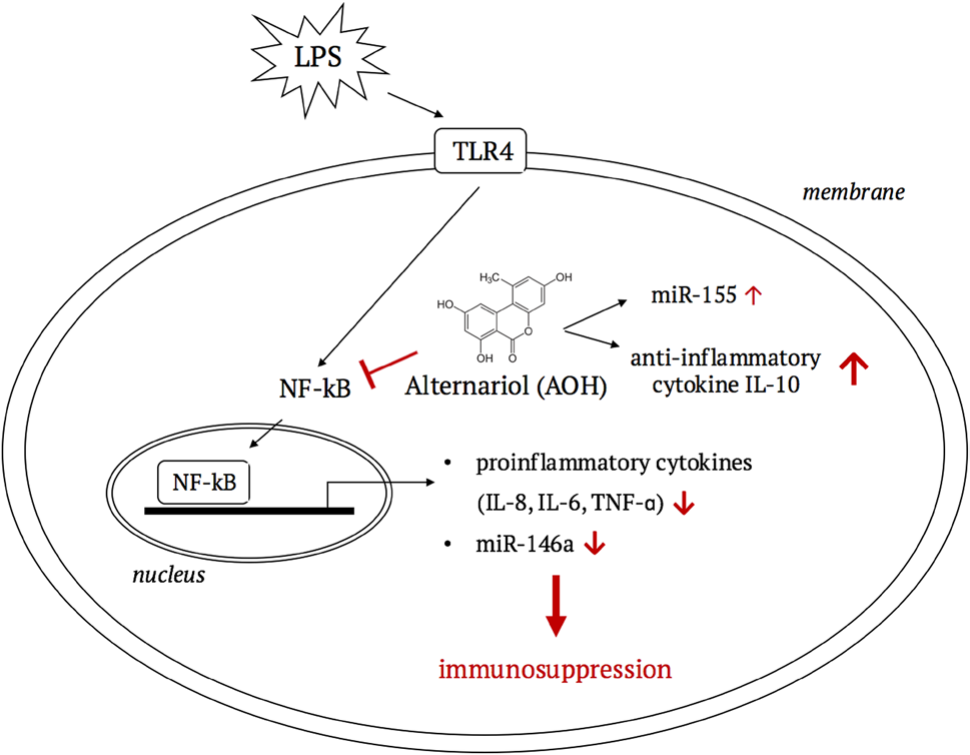
The involvement of NF-κB in AOH-mediated immunosuppression. A suggested cellular pathway illustrating the consequences of AOH-induced NF-κB suppression on cytokine and miRNA expression resulting in a suppressed immune response

Reduced levels of proinflammatory cytokines though, correlate well to the previously observed AOH-induced NF-κB suppression and suggest that AOH-induced decrease of IL-6, TNF-α and IL-8 in stimulated macrophages most likely arise from NF-κB suppression (Fig. 6). Diminished TNF-α secretion was also observed in AOH (15 µM) exposed LPS-stimulated THP-1 cells in another study and attenuated secretion of IL-8 and IL-6 was furthermore reported in BEAS-2B and RAW264.7 macrophages after AOH (5–10 µM) exposure (Grover and Lawrence 2017; Solhaug et al. 2016).

In the absence of a LPS stimulus, AOH did not affect basal NF-κB activity (data not shown). Thus, as expected, AOH did not modulate basal transcript levels of IL-8 and IL-6, which is in line with previous findings (Fig. 3c, d) (Solhaug et al. 2015). Although, TNF-α transcription was significantly (*p* < 0.05) reduced by AOH (20 µM) in non-stimulated macrophages after 5 h incubation, no impact could be observed anymore with increasing exposure period (Fig. 3c, d). In LPS-stimulated macrophages, the cytokine transcription profile after 5 h of exposure correlated well with the cytokine secretion levels after 20 h; however, we observed distinct differences in mRNA levels at different time points (Fig. 3a, b). While AOH downregulated IL-8 transcription at both time points (5 and 20 h), TNF-α transcription was only downregulated after 20 h and the impact on IL-6 transcription was inversed from significant downregulation after 5 h to a significant upregulation after 20 h of exposure. Downregulation of LPS-induced TNF-α transcription is in line with recent studies; however, Grover and Lawrence (2017) found a significant downregulation of IL-6 transcription in AOH (10 µM) exposed LPS-stimulated BEAS-2B cells (Grover and Lawrence 2017; Solhaug et al. 2016). The IL-6 mRNA levels though, were measured after 24 h of exposure, as opposed to 20 h in the present study.

Since miRNAs are known as post-transcriptional regulators of cytokine genes, we suspected them to be involved in the modulation of IL-6 and TNF-α transcription. So far, only miRNAs (miR-34 family and miR-29a) potentially involved in AOH-induced cell cycle arrest and p53 induction have been identified (Solhaug et al. 2012; Vejdovszky et al. 2017b). Thus, in addition to miR-125b, we furthermore determined transcription levels of miR-16, miR-146a and miR-155, which are involved in the regulation of TLR/NF-κB signalling and NF-κB target genes (O’Neill et al. 2011). miR-125b and miR-16 are both directly targeting TNF-α transcripts thus preventing translation (Jing et al. 2005; Tili et al. 2007). However, AOH did not significantly affect the transcript levels of both miRNAs. Furthermore, miR-125b and miR-16 did not respond to the LPS stimulus in THP-1 macrophages, showing similar relative transcript levels for the solvent control and LPS treated cells (Fig. 5). Thus, miR-125b and miR-16 may be regulated by another pathway than the LPS/TLR4 signal transduction pathway in differentiated THP-1 macrophages. miR-146a on the other hand, was significantly downregulated by AOH (2–20 µM) in a concentration-dependent manner, whereas LPS induced a significant increase of miR-146a compared to the solvent control (Fig. 5). This may indicate a potential link between miR-146a expression and AOH-induced suppression of NF-κB. So far, miR-146a was identified a LPS-responsive miRNA, which is expressed in a NF-κB-dependent manner and negatively regulates inflammation by targeting various molecules (Taganov et al. 2006). This is in line with our findings, indicating that miR-146a transcription most likely decreased as a consequence to suppressed NF-κB activation induced by AOH.

Besides targeting central proteins involved in NF-κB signalling (e.g., TRAF-6 and IRAK-1), miR-146a was furthermore reported to negatively regulate IL-6 translation in previous studies (He et al. 2014). Reduced levels of miR-146a would therefore suggest upregulation of IL-6 transcription, which we indeed observed in LPS-stimulated macrophages exposed to AOH for 20 h (Fig. 3b**)**. In contrast, after 5 h exposure IL-6 transcription was significantly downregulated by AOH. This may be explained by the fact, that miR-146a is a delayed-response gene, reaching a peak after 24 h post stimulation (Saba et al. 2012). Thus, it may be speculated that IL-6 transcription was suppressed as a consequence of NF-κB repression in the first place, and increased at a later time point due to reduced miR-146a transcription. Similar to miR-146a, miR-155 was also found to be induced through LPS/NF-κB activation, mainly exerting its function by suppressing negative regulators of inflammation (Gatto et al. 2008). However, in the present study the contrary was observed. miR-155 was not induced in THP-1 derived macrophages by LPS, but instead by AOH (20 µM), which suppressed LPS-induced NF-κB activation. Therefore, alternative pathways have to be considered being involved in the impact of AOH on miR-155 transcription.

IL-10 is an anti-inflammatory cytokine with pleiotropic functions in immunoregulation, such as inhibition of proinflammatory cytokine expression, downregulation of NF-κB activity and TLR signalling (Mocellin et al. 2003). The immunosuppressive effects induced by AOH so far, are further underlined by the impact on the anti-inflammatory cytokine IL-10. The expression of IL-10 is known to be induced by various endo- and exogenous stimuli, including the NF-κB transcription factor (Saraiva et al. 2005). Thus, suppression of NF-κB would consequently suggest a reduced expression of IL-10. Surprisingly however, we found a significant increase of both mRNA and secretion levels of IL-10 in LPS-stimulated THP-1 derived macrophages exposed to 20 µM AOH, indicating that AOH most likely triggered further signalling pathways promoting an increase of IL-10 expression (Figs. 3a, b, 4). This hypothesis is additionally supported by induced IL-10 mRNA levels in non-stimulated macrophages after AOH (20 µM) exposure (Fig. 3c, d**)**, despite lacking activation of NF-κB (data not shown). To the best of our knowledge, this is the first study demonstrating impact of AOH on IL-10 transcription. However, contrary to our data, secretion has not been found to be affected by AOH in other cell lines (RAW264.7 macrophages, BEAS-2B), which may be attributed to cell specifity (Grover and Lawrence 2017; Solhaug et al. 2015). Besides NF-κB, IL-10 induction is furthermore linked to the transcription factor AP-1 and p38/ERK signalling pathway (Patel et al. 2012). In addition, increased levels of IL-10 may potentially be a consequence of AOH-induced expression of the tumor suppressor protein p53, since this protein was previosuly reported to induce IL-10 to suppress inflammation and inhibit macrophage functions (Zheng et al. 2005). Increased levels of p53 have been linked to suppression of the mammalian target of rapamycin (mTOR) signalling pathway as a response to AOH-induced DNA damage followed by increased autophagy and senescence in RAW264.7 macrophages exposed to AOH (15–30 µM for 24 h) (Solhaug et al. 2014). The mTOR pathway possesses a broad range of functions and besides inducing cell survival and proliferation it was also found to regulate the immune response by inducing NF-κB, which can be repressed by the immunosuppressant rapamycin (Dan et al. 2008). These findings correspond to our data and indicate that the observed AOH-induced NF-κB suppression followed by reduced secretion of proinflammatory cytokines in this study may potentially arise from p53-induced downregulation of mTOR caused by DNA damage.

In conclusion, this study provides further evidence for the immunosuppressive properties of the mycotoxin AOH. Under proinflammatory conditions, in THP-1 derived macrophages AOH exerted its immunosuppressive effects by downregulation of proinflammatory cytokines (IL-8, IL-6 and TNF-α) via suppressing the NF-κB signalling pathway and altering NF-κB-dependent regulatory miRNAs. This effect is further underlined by enhanced IL-10 gene expression by AOH, even in absence of an inflammatory stimulus. The observed effects were mainly induced by comparably high AOH concentrations (2 and 20 µM), giving rise to the question, whether such concentrations are physiologically achievable. Studies on in vivo exposure are still scarce. In NMRI mice systemic bioavailability of AOH after oral application has been reported to be marginal (< 0.1% of the applied dose), with about 90% being eliminated via feces and around 9% subjected to a rapid metabolism to metabolites excreted via urine (Schuchardt et al. 2014). These data indicate that in vivo concentrations of AOH in the higher micromolar range are unlikely to be reached in the bloodstream. Nevertheless, it cannot be excluded that respective concentrations might be achieved locally in the gastrointestinal tract where, among other cell populations, epithelium-associated macrophages might indeed be affected thus potentially triggering the innate immune response.

It is still a long journey to fully elucidate the comprehensive complex pattern of physiological mechanisms that are mediated by AOH, nevertheless, the present study provides new insights into the interaction of AOH with critical regulators involved in innate immune response regulation such as the NF-κB pathway and regulatory miRNAs, thereby providing the basis for subsequent in-depth studies.

## Abbreviations

AOH: Alternariol
ROS: Reactive oxygen species
miRNA: MicroRNA
ER: Estrogen receptor
LPS: Lipopolysaccharide
NF-κB: Nuclear factor kappa B
TRAF-6: TNF receptor-associated factor 6
IRAK-1: Interleukin-1 receptor-associated kinase 1
TTC: Threshold of toxicological concern
